# iCover as Bridging Stent Graft in Fenestrated Endovascular Aortic Aneurysm Repair

**DOI:** 10.1177/15266028241270862

**Published:** 2024-09-06

**Authors:** Anna-Leonie Menges, Vincent Landré, Lorenz Meuli, Alexander Zimmermann, Benedikt Reutersberg

**Affiliations:** 1 Department of Vascular Surgery, University Hospital Zurich, University of Zurich, Zurich, Switzerland

**Keywords:** bridging stent graft, fenestrated endovascular aneurysm repair, FEVAR, iCover stent graft, technical success, endoleak, in-hospital mortality

## Abstract

**Background:**

Complex endovascular repair with fenestrated or branched stent grafts is a common approach for treating various types of aortic aneurysms. Bridging stent grafts (BSs) are crucial in connecting aortic endoprosthesis to target vessels, yet current options have demonstrated significant complications.

**Objective:**

This retrospective single-center study evaluates the initial outcomes and durability of the iCover stent graft (iCover-SG) when used as a BS in fenestrated endovascular aneurysm repair (FEVAR).

**Methods:**

Retrospective analysis screened procedures for complex aortic aneurysms between August 2021 and January 2024. Patients who underwent FEVAR with iCover-SG as BS were included. Primary and secondary endpoints focused on freedom from iCover-SG-related target vessel instability, technical success, and postoperative outcomes.

**Results:**

Within the cohort of 28 patients, 94 iCover-SGs were used as BS, supplying 87 target vessels. The freedom from iCover-SG-related target vessel instability throughout the study reached 94% (82/87). Technical success rates were notably high, with primary success achieved in 94% of cases and secondary success in 99%. Over the follow-up duration, there were instances necessitating reintervention related to iCover-SG, including 4 cases of endoleak, 2 cases of T1cEL, and 2 cases of T3cEL. In-hospital mortality was 7% (n=4), with 2 cases attributed to intraoperative complications. Importantly, no deaths were directly attributed to iCover-SG-related issues.

**Conclusion:**

The iCover-SG demonstrates promising initial outcomes as a BS in FEVAR, with high technical success rates and satisfactory rates of target vessel instability. Continued monitoring and further studies are warranted to assess long-term durability and outcomes.

**Clinical Impact:**

This study shows that the iCover stent graft achieves satisfactory technical success and target vessel stability in the short- and mid-term when used as a bridging stent graft in FEVAR procedures. Its successful integration into clinical practice broadens the range of available options, providing clinicians with more versatile tools for managing complex endovascular aortic aneurysms. This expanded selection of bridging stent grafts allows for more personalised treatment strategies, improving procedural precision and patient outcomes. The iCover stent graft’s reliable performance highlights its potential as a valuable addition to current endovascular techniques, ultimately enhancing patient care in challenging cases.

## Introduction

Complex endovascular repair with fenestrated or branched stent grafts is widely used in treating thoracoabdominal, pararenal, juxtarenal, or iliac artery aneurysms.^1,2^ In these procedures, an aortic endoprosthesis must be connected to the corresponding target vessels with stent grafts, called bridging stent grafts (BSs). A few different BSs are available on the market, varying from self-expanding to balloon-expanding. None of these are approved for this purpose and are generally not dedicated for use as BS in the fenestrated or branched endovascular aneurysm repair (F/BEVAR) technique. The BSs used so far have shown several complications, such as dislocations, stent fractures, occlusion, and endoleaks during follow-up.^3–10^

An ideal BS is characterized by its visibility, flexibility, good radial strength, and small profiles, allowing for easy insertion through small sheaths. It should also resist external influences, such as arteriosclerotic plaques or aortic movement, seal effectively, and remain patent in the long term.

The iCover stent graft (iCover-SG; iVascular, Barcelona, Spain), which became available in 2021, promises greater flexibility with excellent navigability and better visualization. The question is whether this will address some difficulties better than the current available BS. This single-center study analyzed a series of all-comers with fenestrated endovascular aneurysm repair (FEVAR) treated at a university referral center since the introduction of the iCover-SG and aims to assess the initial outcomes and durability of iCover-SG when used as BS.

## Patients and Methods

This retrospective data analysis screened all procedures for complex aortic aneurysms between August 2021 and January 2024 for eligibility.

Patients were included if iCover-SG were deployed as a BS during a FEVAR procedure. Excluded were patients who underwent FEVAR without using iCover-SG, patients who received iCover-SG for other vascular pathologies than described above, and those who withdrew or declined the institutional general consent on observational research.

Data acquisition was following the Declaration of Helsinki. Written informed consent was obtained from all patients, or their enrollment was provided according to the Federal Human Research Act in case of a missing consent statement. The regional research ethics committee approved this analysis ethically (BASEC Nr.2023-00047).

### Data Collection

Patients were identified through the institutional clinical information system (KISIM 5.1.0.3; CISTEC AG, Zurich). Baseline clinical data included sex, age, comorbidities, and antiplatelet or anticoagulation therapy.

Perioperative and postoperative outcomes were evaluated by characterizing the implanted aortic endoprosthesis and assessing intraoperative events, postoperative complications (cardiac, respiratory, renal, gastrointestinal, wound healing, puncture site complications), the appearance of endoleak, reinterventions, and mortality.

Two reviewers analyzed postoperative computed tomography angiograms (CTAs) using a 3-dimensional workstation (XERO Viewer 8.1.2; Agfa HealthCare N.V., Mortsel).

### Primary Endpoints

The primary endpoints are freedom from iCover-SG-related target vessel instability as the safety endpoint and technical success as the endpoint for efficacy. Technical success was also subdivided into primary, assisted primary, and secondary technical success. This was evaluated related to the iCover-supplied target vessels.

Both are defined according to Oderich et al’s^11^ reporting standards for endovascular aortic repair of aneurysms involving the renal-mesenteric arteries, which are described in detail under definitions.

### Secondary Endpoints

Secondary endpoints consisted of clinical in-hospital and early midterm outcomes. These included major adverse events, not iCover-SG-related reinterventions or secondary procedures, and not iCover-related morbidity and mortality. Given the focus on iCover-SG, issues and complications related to other BS were described but excluded from the iCover-SG evaluation and described in secondary endpoints.

### Definitions

The outcomes were defined following the current reporting standards for endovascular aortic repair of aneurysms involving the renal-mesenteric arteries of Oderich et al.^11^ Accordingly, freedom from iCover-SG-related target vessel instability is defined as “any death or rupture related to side branch complication (eg, endoleak [EL], rupture) or any secondary intervention indicated to treat a branch-related complication, including endoleak, disconnection, kink, stenosis, occlusion, or rupture.”^11,12^

The primary technical success was assessed using an “intent-to-treat” approach by successfully introducing and deploying the iCover-SG without mortality, T1cEL/T3cEL, branch occlusion, graft limb obstruction, or the need for surgical conversion. Assisted primary or secondary technical success was considered to describe any unplanned endovascular or surgical procedures required during treatment.^11^

Major adverse events were defined as death or any major complications that result in an escalating level of care or severe disability, all-cause mortality, myocardial infarction, respiratory failure requiring prolonged (>24 hours from anticipated) mechanical ventilation or reintubation, renal function decline resulting in >50% reduction in baseline of estimated glomerular filtration (eGFR) or new onset of dialysis, bowel ischemia which required bowel resection, major stroke or paraplegia, mortality rates, and the need for reinterventions. These events were monitored throughout the in-hospital stay and follow-up period.^11^ Renal insufficiency was defined as an eGFR below 60 mL/min, and renal impairment was characterized by a postoperative decrease in eGFR of more than 50% or a 2 to 3 times increase in serum creatinine (sCr) levels compared with the preoperative eGFR (RIFLE-criteria).^12,13^

### Postoperative Treatment and Follow-up Protocol

All patients received postoperatively dual antiplatelet therapy within our local treatment standard for at least 6 weeks. Clopidogrel was added to preexisting anticoagulants for 6 weeks and switched to anticoagulants plus acetylsalicylic acid if the patient was anticoagulated before.

For abdominal aortic aneurysm (AAA), a contrast-enhanced ultrasound (CEUS) was performed before discharge, after 6 months, and with unremarkable findings annually after the first year. Usually, 1 CTA was performed 4 to 6 weeks postoperatively or if CEUS showed an endoleak or aneurysm sac expansion. For thoracoabdominal aortic aneurysms, a CTA was performed before discharge, after 6 months and after 1 year annually.

### Stent Graft Design

**Figure 1.**
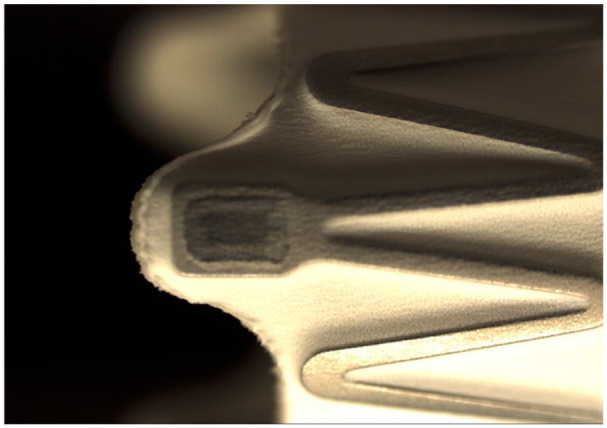
iCover stent graft and radiopaque tantalum marker.

**Figure 2.**
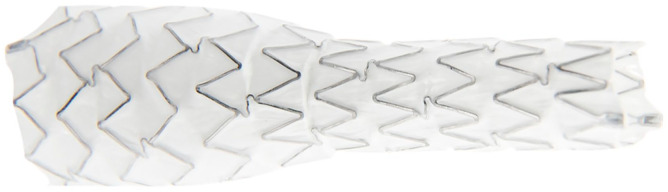
Example of an iCover stent graft. Demonstration of flexibility in diameters through its open-cell design with alternating connection bridges.

The iCover-SG is a balloon-expandable stent graft of Cobalt Chromium alloy covered with ePTFE internally and externally. The stent is manufactured by laser-cutting metal tubes. It has 3 radiopaque tantalum markers at each end (Figure 1). Its open-cell design allows adaptation to different artery diameters with alternating connection bridges (Figure 2). The covered stent system is preassembled in a positioning system, including a double-lumen coaxial balloon catheter. The iCover-SG is available in various diameters (5, 6, 7, 8, 9, 10 mm) and lengths (17, 27, 37, 57 mm). It fits through 6 French sheaths (5, 6, 7 mm) or 7 French sheaths for all stents ≥8 mm in diameter, except for the 8 × 17 mm stent that fits a 6F sheath. One French size larger than indicated on the label is advisable if long or braided introducers are used. It is designed for a 0.035 inch (0.89 mm) wire, and the catheter length is 80 or 140 cm.^14^

### Statistical Analysis

Categorical variables are presented with counts and percentages. Continuous variables are summarized by means and standard deviation if normally distributed. Skewed continuous variables were summarized by median and interquartile range (IQR) or median and minimum to maximum, depending on sample size.

## Results

### Patients and Procedural Details

Between August 2021 and January 2024, a total of 175 iCover-SGs were implanted in 63 patients for different indications. Ninety-four iCover-SGs were implanted in 87 target vessels with fenestrated endografts. In 7 of these cases, the BS was too short and was extended to ensure safe sealing. In addition, 8 target vessels were bridged with stent grafts of other manufacturers due to stock shortages (eVentus, Advanta).

Of the 94 iCover-SGs, 45 were deployed in visceral (coeliac trunk, n=19, superior mesenteric artery, n=26) and 49 in renal arteries. The mean age of these patients was 75±10 years, and 25 were men. Twelve patients were treated with FEVAR for type 1a endoleak (T1aEL) after infrarenal endovascular aortic aneurysm repair (EVAR) to achieve proximal sealing, 11 patients were treated for juxtarenal, 2 for pararenal, 1 for suprarenal, and 2 for thoracoabdominal aortic aneurysms (Table 1).

**Table 1.** Demographics, Preoperative Parameters, and Anatomical Characteristics.

	Total (N=28)
Demographics	
Age (years) mean±SD	75±10
Sex (male)	25 (89)
Comorbidities	
Arterial hypertension	21 (75)
Ever smoker	15 (54)
Statin use	16 (57)
Diabetes mellitus	6 (21)
Coronary artery disease	8 (29)
Chronic kidney disease	5 (18)
COPD	7 (25)
Previous operations	
Prior aortic surgery	
Thereof prior thoracic endograft	2 (7)
Thereof prior abdominal endograft	12 (43)
Indications and anatomical characteristics	
Endoleak type Ia after EVAR	12 (43)
Juxtarenal aortic aneurysm	11 (39)
Pararenal aortic aneurysm	2 (7)
Suprarenal aortic aneurysm	1 (4)
Thoraco-abdominal aneurysm (Crawford)	
Type 2	1 (4)
Type 5	1 (4)
Preoperative AA diameter (mm), mean±SD	70±19
Coagulation on admission	
APT	22 (79)
Dual APT	1 (4)
Anticoagulation	2 (7)
Anticoagulation and APT	0 (0)

Results are given as numbers (%), mean±standard deviation (SD).

Abbreviations: AA, aortic aneurysm; APT, antiplatelet therapy; COPD, chronic obstructive pulmonary disease.

The mean diameter of the aortic aneurysms was 70±19 mm. 14 patients had prior aortic surgery or endovascular aortic intervention.

Detailed patient characteristics, endograft, and procedural details are shown in Tables 1 and 2.

**Table 2.** Procedural Details.

	Total (N=28)
Mainbody	
Cook Medical	11 (39)
Thereof physician modified	6 (21)
Terumo Aortic (*FEVAR, Anaconda*)	17 (61)
Number of target vessels with iCover-SG	87
Number of implanted iCover-SG	94
Number of BS of other manufacturer	8
Number of fenestrations	95
1 fenestration	1
2 fenestrations	3
3 fenestrations	8
4 fenestrations	16
Length of procedure (min), median (Q1, Q3)	190 (161, 239)
Dose area product (Gy.cm^2^), median (Q1, Q3)	466 (212, 686)
X-ray duration (min), median (Q1, Q3)	57 (43, 75)

Results are given as numbers (%) and median with 1. and 3. quartile.

Abbreviations: BS, bridging stent graft; FEVAR, fenestrated endovascular aortic repair; SG, stent graft.

### Primary Endpoints

In our cohort, a total of 95 iCover-SGs were deployed. Of these, 94 were successfully implanted as BS, with 1 iCover-SG dislodged from the balloon during unprotected advancement into the target vessel.

The iCover-related freedom from target vessel instability was 94% (82/87). This was observed alongside the following events: in 1 patient, perforation or rupture occurred during renal artery cannulation and pre-dilatation for stenosis, resulting in hemodynamically significant bleeding that necessitated control via occlusion of the renal artery with a vessel plug. This patient passed away due to multi-organ failure (MOF) during the hospital stay and is described in detail under “Secondary endpoints.”

The early midterm follow-up noted 3 newly detected T1cELs and 2 T3cELs. Two T1cEL patients required treatment on day 63 and day 105, while the third underwent surveillance due to the small size of the T1cEL following a multidisciplinary meeting discussion. The patients with T3cEL were treated on days 140 and 308. All 4 endoleaks were effectively treated by extending or relining the iCover-SG, and the T1cEL under surveillance was resolved after 6 months.

No other iCover-related target vessel instability was noted during the follow-up, especially no iCover-SG-related rupture or mortality.

The primary and assisted primary technical success rate was 94% (82/87), with a secondary technical success rate of 99% (86/87). In addition to the dislocated iCover-SG and perforated and plugged renal artery mentioned above, technical failures included 2 patients in whom antegrade cannulation of 3 target vessels was not feasible (1 renal artery, 1 celiac trunk, 1 superior mesenteric artery). In these cases, target vessel cannulation was achieved through retrograde puncture following open surgical conversion. The implantation of the iCover-SG for all target vessels was accomplished in both patients via antegrade deployment after establishing a through-and-through wire.

Table 3 provides further details regarding procedural events and outcomes associated with iCover-SG. Notably, additional relining of iCover-SG with nitinol stents was performed in 6 cases, 4 of which were due to target vessel dissection and 2 for reinforcement of the iCover-SG due to kinking or mild stenosis. In one other case, accidental perforation of a renal artery occurred during cannulation, but iCover-SG deployment successfully sealed the perforation site. While these observations lie outside current definitions of technical success and target vessel instability, they are worth reporting.

**Table 3.** iCover-Related Outcomes.

	Number of target vessels, n=87
Total implanted iCover-SG (n)	94
Intraprocedural and in-hospital outcome	
Intraprocedural events and complications	
iCover-SG events	
iCover-SG misplacement during deployment	1 (1)^a^
Additional covered stent graft	5 (6)^b^
Additional nitinol stent	5 (6)
Open surgery and direct puncture of target vessel	3 (3)^a^
Target vessel events	
Vessel perforation	2 (2)
Vessel dissection	4 (5)
Vessel occlusion/ intentional iCover-SG occlusion	1 (1)^a, c, d^
Technical success	
Primary and assisted primary technical success, %	**94**
Secondary technical success, %	**99**
iCover-related reinterventions	
For type 1c endoleak	0 (0)
For type 3c endoleak	0 (0)
For iCover-SG stenosis	0 (0)
For iCover-SG occlusion	0 (0)
Short-/ midterm outcome	
iCover-related complications	
Occlusion	0 (0)
Stenosis	0 (0)
Kink	0 (0)
Dislocation	0 (0)
Type 1c endoleak	3 (3)
Type 3c endoleak	2 (2)
iCover-related reinterventions	
For type 1c endoleak	2 (2)^c,e^
For type 3c endoleak	2 (2)^c^
For iCover-SG stenosis	0 (0)
For iCover-SG occlusion	0 (0)
Overall iCover-related rupture	0 (0)
Overall iCover-related mortality	0 (0)
Freedom of iCover-related target vessel instability, %	**94**

Results are given as numbers (%).

Abbreviation: iCover-SG, iCover stent graft.

a Counts to technical success.

b Additional stent graft for sealing.

c Counts to target vessel instability.

d Patient in hospital deceased, bold: primary endpoints.

e One type 1c endoleak is under surveillance at this time.

### Secondary Endpoints

The median length of in-hospital stay for the study cohort of these 28 patients was 7 days (IQR: 5–11), with a median follow-up duration of 10 months (IQR: 3–17).

During the hospital stay, 6 patients developed major adverse events. Acute kidney injury and respiratory failure occurred in 4 cases each, while MOF was observed in 2 cases, with 1 of them also experiencing mesenteric ischemia (Table 4).

**Table 4.** In-Hospital and Early Midterm Clinical Outcome.

	Total, N=28
Follow-up (month), median (Q1, Q3)	10 (3, 17)
In-hospital outcome	
Major adverse events (n=6)	
Acute renal failure/permanent dialysis	4/0 (14/0)
Myocardial infarction	0 (0)
Mesenteric ischemia	1 (4)^a^
Respiratory failure	4 (14)
Spinal cord ischemia	0 (0)
Multi-organ failure	2 (7)^a^
Secondary procedures (n=6)	
For hemorrhage	2 (7)
For access complications	3 (11)
For emboly complications	0 (0)
For bowel ischemia	1 (4)
For BS occlusion^b^	1 (1)^a^
For exploration	1 (4)
In-hospital-mortality	2 (7)
Hospital length of stay (d), median (Q1, Q3)	7 (5, 11)
Short-/early midterm outcome^c^	
Overall mortality	4 (14)
Aneurysm-related mortality	0 (0)
Reinterventions^d^ (n=6)	
For type 1 endoleak	3 (11)^d^
For type 2 endoleak	0 (0)
For type 3 endoleak	0 (0)
For access complications	2 (7)
For emboli complication	0 (0)
For other aortic endograft-related reasons	1 (4)

Results are given as numbers (%) and median with 1. and 3. quartile.

Abbreviation: BS, bridging stent graft.

a Patient in hospital deceased.

b Another than iCover.

c Outcome after discharge.

d Not iCover-related type 1c/3c endoleak interventions.

Both cases resulted in MOF following intraoperative hemorrhage. The first case involved the patient, who was mentioned previously, who suffered renal artery perforation while deployed. Shortly after the operation, the patient developed lactic acidosis and significant systemic inflammatory response syndrome (SIRS). Despite exploratory laparotomy revealing no bowel ischemia and normal stent graft perfusion, the patient succumbed to MOF on the first postoperative day and passed away. The second case involved a patient with an accidental rupture of the external iliac artery during the procedure, leading to hemodynamic instability, which required immediate conversion and open reconstruction of the external iliac artery. This patient required early reintervention on the first postoperative day for an early BS occlusion (other than iCover-SG) of the renal artery due to dissection. The attempts to recanalize the renal artery failed.

In addition, this patient developed severe bowel ischemia necessitating extensive bowel resection despite all other stent grafts appearing patent, ultimately succumbing to MOF on the third postoperative day. Both were most likely related to different factors with cholesterol micro embolizations, hypotensive episodes, and high requirement of catecholamines. Unfortunately, the relatives declined an autopsy so that it could not be conclusively clarified. These 2 cases contributed to an overall in-hospital mortality rate of 7%.

Four other patients required secondary interventions during their hospital stay: 2 of them for retroperitoneal hematoma after conversion and direct puncture of the target vessel, and the other 2 were for access-related issues such as wound infection and new onset of short-distance claudication due to high-grade common femoral artery stenosis.

During follow-up, reinterventions were performed in 6 other patients, including interventions for T1cEL (n=2) and T3cEL (n=2), as described previously. In addition, 2 cases necessitated reintervention for T1bEL, and 1 proximal extension for T1aEL with an aortic cuff was performed. No reinterventions for other endoleak types were reported.

In another case, a presentation consistent with Mid Aortic Syndrome was evident, characterized by significant stenosis of the aortic stent graft prosthesis, as the fabric of the aortic stent graft (Anaconda, Terumo aortic (Inchinan, UK)) failed to fully expand, with fenestrations for both renal arteries and the superior mesenteric artery almost at the same level. Due to short-distance claudication and lack of improvement, the patient underwent aorto-iliac bypass from the descending aorta to revascularize the lower extremity after 4 months. Furthermore, 2 patients passed away during follow-up; both were not aortic- or BS-related, resulting in an overall mortality of 14% (n=4; Table 4).

## Discussion

This study investigated the reliability of iCover-SG as BS in complex endovascular aortic aneurysm repair. The iCover-SG showed comparable results with other stent grafts used as BS regarding freedom of target vessel instability and technical success in a highly challenging patient cohort with different endoprosthesis in the short and early midterm.^3–6,10,15^

The essential endpoint of this study is the combined endpoint of “freedom from target vessel instability.” This especially described the outcome for bridging stents and target vessels. In general, a stable target vessel is crucial for the long-term success of the procedure as it reduces the risk of adverse events and potential complications.^16^

This study’s overall iCover-SG-related freedom of target vessel instability was 94%. The main issues for the missing 6% during the follow-up were T1cEL and T3cEL. In this cohort, 4 cases required reinterventions for endoleak, 2 for T1cEL and 2 for T3cEL. This could be effectively resolved by extending and relining the BS. The outcome of the iCover-SG is in line with the results published on other BS. Also, Mastracci et al reported that T3cEL was the primary cause of reintervention following complex endovascular repair, and the meta-analysis by Nana et al reported an incidence of T3cEL ranging from 1% to 3%.^3,17^ In their meta-analysis including 1406 target vessels, the freedom of target vessel instability reached 91% at 6 to 36 months, and in a systematic review by Mezetto et al,^7^ with 9556 target vessels, the freedom of target vessel instability was 96% at 12 to 35 months.^10^ Considering recently published results for balloon-expandable stent grafts from other manufacturers, the iCover-SG demonstrates comparable outcomes. For both the BeGraft (Bentley InnoMed GmbH, Hechingen, Germany) and the V12/iCast (Getinge AB, Göteborg, Sweden), the freedom of target vessel instability at 1 year is 98%,^9,15^ for the Viabahn/VBX (W.L. Gore & Associates, Inc, Newark, Delaware, USA) at 6 months it is 92%,^6^ and 91% for the LifeStream at 6 and 12 months (BD, Becton, Dickinson and Company, Franklin Lakes, New Jersey, USA).^4^

The primary and assisted primary technical success rates were 94%, indicating that most iCover-SG were effectively deployed and could be positioned in the intended anatomical sites. The secondary technical success, which evaluates the stent graft’s ability to be successfully placed through procedural extension, was achieved in 99% of cases. The iCover-SG could be deployed in visceral, renal, and other branches. This also highlights the versatility and applicability of a bridging stent in various anatomical locations, supported by the availability of different sizes for the iCover-SG.^14^ However, the technical success rate must be viewed critically to describe the applicability of a bridging stent. Hence, it encompasses many technical steps, not all primarily related to the BS. Technical success includes, by definition, the successful cannulation of the target vessel. Thus, complications arising at this stage, such as perforation, count toward this endpoint, although the BS has not yet played any role. However, in terms of intention-to-treat methods and to provide comparable endpoints for the entire procedure, we opted to define the endpoint based on the reporting standards for endovascular aortic repair of aneurysms involving the renal-mesenteric arteries set by Oderich et al^11^ and thus not only specific for the iCover-SG. In our analysis, there was only 1 technical issue directly related to the iCover-SG. It was when the iCover-SG inadvertently detached from its delivery system due to sheath-related issues. The other problems primarily occurred during the cannulation of the target vessel. Two renal arteries had to be sealed due to hemodynamically significant bleeding after perforation, and 3 target vessels required open surgery for a retrograde puncture for successful cannulation. In these 5 cases, the stent graft had yet to be involved. Finally, of 95 deployed iCover-SGs, 94 could be successfully placed. This demonstrates satisfactory results of the iCover-SG in achieving technical success. Based on the author’s experience in the case of FEVAR, BEVAR, or other indications, one should implant the iCover-SG sheath-protected to prevent slipping off the balloon and subsequent misplacement.

**Figure 3.**
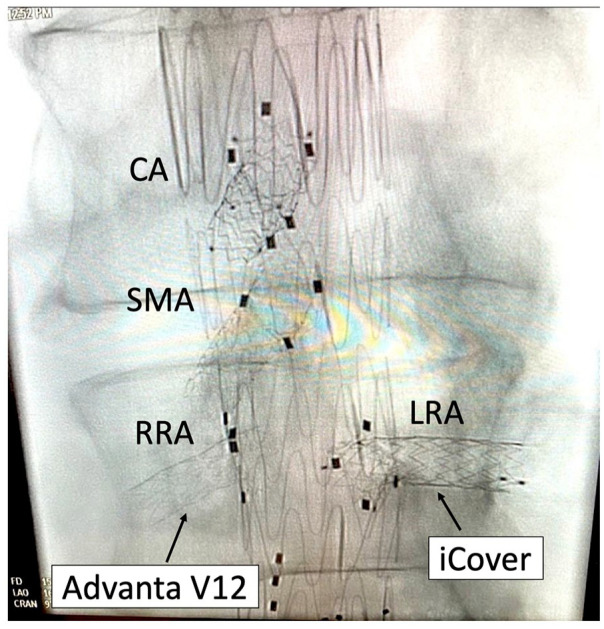
Visualization of an iCover stent graft compared to an Advanta/iCast stent graft. CA, coeliac artery; SMA, superior mesenteric artery; RRA, right renal artery; LRA, left renal artery.

One of the notable advantages of the iCover-SG, especially from a technical standpoint, is its excellent visibility, even in obese patients. This is exemplified in Figure 3, where various stent grafts have been implanted in the same patient, showcasing the noticeable differences in visibility.

Regarding existing literature concerning the results of other BSs, which typically report technical success rates ranging from 83% to 100%,^3–8,10^ the technical success rate is comparable.

Also, the primary and secondary patency rates of 98% are promising. The missing 2% arose because of the above-described intentionally sealed renal artery with an Amplatz Plug for bleeding control and the other one for dissection and following occlusion of the target vessel.

Throughout the entire follow-up period, no occlusion of an iCover-SG was observed. However, in multiple studies, BSs in renal arteries have a higher rate of complications and occlusion in the longer term, so this needs further observation.^16,18,19^ Mastracci et al^20^ proposed theoretical postulations that the higher failure rate of renal branches compared with visceral branches in BS may be attributed to factors such as the variable renal angle, greater respiratory motion, smaller vessel size, and higher resistance perfusion. These findings were also confirmed by the systematic review conducted by Mezzetto et al^7^ but could not be demonstrated in our cohort so far. Of note, neither the sample size nor the follow-up length allows any firm conclusions regarding these considerations.

### Limitation

While this retrospective observational single-center cohort study has inherent limitations, it offers valuable clinical insights into real-world patients treated as all-comers in a high-volume referral center. However, the study’s design and the limited sample size increase the risk of drawing flawed conclusions. In addition, the evaluation of outcomes was confined to a median 10-month follow-up period.

Standardized reporting facilitates later comparisons with other studies. Nonetheless, a direct comparison of the iCover-SG with other BSs is essential, ideally in a randomized controlled trial.

## Conclusion

The iCover-SG, when deployed as a BS in FEVAR, demonstrates satisfactory rates of freedom from target vessel instability in the short and early midterm and achieves good technical success. However, the observed complications, such as stent displacement, target vessel perforation, and endoleak development, underscore the importance of optimizing procedural techniques and careful patient selection. Future studies with larger sample sizes and extended follow-up periods are needed to validate these findings and assess long-term durability.
